# Isorhamnetin Ameliorates Non-Esterified Fatty Acid-Induced Apoptosis, Lipid Accumulation, and Oxidative Stress in Bovine Endometrial Epithelial Cells via Inhibiting the MAPK Signaling Pathway

**DOI:** 10.3390/antiox14020156

**Published:** 2025-01-28

**Authors:** Haimiao Lv, Lijuan Liu, Wenna Zou, Ying Yang, Yuan Li, Shengji Yang, Aixin Liang, Liguo Yang

**Affiliations:** 1 Key Laboratory of Agricultural Animal Genetics, Breeding and Reproduction of Ministry of Education, College of Animal Science and Technology, Huazhong Agricultural University, Wuhan 430070, China; lvhaimiao0310@webmail.hzau.edu.cn (H.L.); 2022302110088@webmail.hzau.edu.cn (L.L.); qiuyi@webmail.hzau.edu.cn (W.Z.); hzyy666@webmail.hzau.edu.cn (Y.Y.); li_yuan@webmail.hzau.edu.cn (Y.L.); 149248@webmail.hzau.edu.cn (S.Y.); 2 National Center for International Research on Animal Genetics, Breeding and Reproduction, Huazhong Agricultural University, Wuhan 430070, China

**Keywords:** non-esterified fatty acids, isorhamnetin, endometrial epithelial cells, MAPK signaling, bovine

## Abstract

High concentrations of non-esterified fatty acids (NEFA) in the blood contribute to various metabolic disorders and are linked to endometritis in dairy cows. Isorhamnetin (ISO), a flavonoid found in many plants, is known for its antioxidant, anti-inflammatory, and anti-obesity properties. This study systematically assessed NEFA-induced damage in bovine endometrial epithelial cells (bEECs) and investigated whether ISO alleviates NEFA-induced cell damage and its underlying molecular mechanisms. Our observations revealed that excessive NEFA inhibited proliferation and induced apoptosis in bEECs, accompanied by an increase in the expression of BAX and cleaved caspase-3. We further observed that NEFA could induce lipid accumulation, reactive oxygen species (ROS) generation, and the release of pro-inflammatory factors IL-1β, IL-6, and TNF-α in bEECs. RNA sequencing and Western blot analysis revealed that NEFA induced damage in bEECs by activating MAPK signaling pathway. Notably, ISO treatment ameliorated these effects induced by NEFA, as evidenced by decreased protein levels of BAX, cleaved caspase-3, and PPAR-γ, along with reductions in triglyceride content, ROS generation, and levels of IL-1β, IL-6, and TNF-α. Mechanistically, our experimental results demonstrated that ISO inhibited NEFA-induced activation of MAPK signaling. Overall, ISO shows promise for therapeutic development to address NEFA-related endometritis in dairy cows.

## 1. Introduction

Ketosis is a common metabolic disease that occurs in high-producing dairy cows during early lactation and is associated with the incidence of endometritis [1,2,3,4]. Dairy cows experience a negative energy balance (NEB) due to the increased nutrient demands of lactation and a decrease in dry matter intake (DMI) during the postpartum transition period [5,6]. To compensate for the energy shortage in early lactation, the body elevates fat mobilization, resulting in elevated concentrations of non-esterified fatty acids (NEFA) in the blood. Previous research has indicated that serum NEFA levels in subclinical ketosis cows can reach approximately 0.6 mM [2,7,8]. By comparison, NEFA concentrations can reach approximately 1.2 mM in clinical ketosis or fatty liver [7,9,10,11]. When the antioxidant defenses in these animals are insufficient to counteract the generation of ROS and reactive nitrogen species (RNS) triggered by elevated NEFA levels, these free radicals can adversely affect physiological processes, leading to cellular damage and dysfunction [12]. Recent studies have shown evidence that high concentrations of NEFA (1.2 mM), similar to the NEFA levels observed in dairy cows experiencing fatty liver during the transition period, induce apoptotic cell death in hepatocytes through triggered mitochondrial dysfunction in bovine hepatocytes [10]. Additionally, the high levels of NEFA can lead to the accumulation of lipids and mitochondrial dysfunction in bovine mammary epithelial cells [13,14,15]. Metabolic diseases, including ketosis, can adversely affect the reproductive performance of dairy cows. Recent studies have shown that exposure to high levels of NEFA in vitro leads to fatty acid accumulation in preantral follicles and induces inflammatory responses in the ovarian cortex [16]. Elevated NEFA levels in follicular fluid impair follicular growth in cattle [17]. Furthermore, NEFA induces granulosa cell apoptosis in cattle through the PI3K/AKT/FoxO1 pathway activated by ROS [18]. However, the effect and precise mechanism of high levels of NEFA on the bovine endometrial epithelial cells remain unclear.

Isorhamnetin (ISO), also recognized as 3-O-methylquercetin, is a flavonoid that occurs naturally in various vegetables and fruits, particularly in *Hippophae rhamnoides* L. and the leaves of *Ginkgo biloba* L. [19,20]. ISO has been reported to exhibit a range of beneficial effects, including antioxidant, anti-inflammatory, anti-tumor, and anti-adipogenic activities [21,22,23,24]. Peroxisome proliferator-activated receptor gamma (PPAR-γ) is a ligand-dependent transcription factor that regulates adipogenesis and energy homeostasis [25]. The transcriptional activation of PPAR-γ in the liver induces the adipogenic program, facilitating the storage fatty acids in lipid droplets, as observed in adipocytes [26]. A recent study reported that dietary ISO acts as PPAR-γ antagonist, providing protective effects against obesity and hepatic steatosis in diet-induced obese mice [27]. Interestingly, recent studies have indicated that circulating NEFA in cattle induces dose-dependent activation of PPARs in various tissue cell types, particularly PPAR-δ and PPAR-γ [28]. Furthermore, Lee et al. demonstrated that isorhamnetin inhibits adipogenesis by downregulating PPAR-γ and C/EBP-α in 3T3-L1 adipocytes [22]. In addition, isorhamnetin mitigates cognitive impairments and neuroinflammation induced by high-fat and high-fructose diets by modulating the NF-κB and MAPK signaling pathways [29]. Nevertheless, the impact of isorhamnetin on NEFA-induced damage and lipid accumulation remains unexplored.

In this study, we aimed to explore the full potential effects and molecular mechanisms of NEFA on apoptosis, lipid accumulation, oxidative stress, and inflammation in bovine endothelial cells (bEECs). Importantly, we sought to examine the protective role of isorhamnetin on NEFA-induced damage in bEECs. Our findings may enhance the applicability of isorhamnetin in improving the health and production performance of transitional dairy cows, thereby significantly reducing veterinary costs and increasing the economic benefits for the dairy industry.

## 2. Materials and Methods

### 2.1. Preparation of Non-Esterified Fatty Acids and Isorhamnetin

A stock solution of NEFA (52.7 mM) was prepared by dissolving a mixture of 2.8 mM palmitoleic acid, 7.6 mM stearic acid, 2.6 mM linoleic acid, 16.8 mM palmitic acid, and 22.9 mM oleic acid (MedChemExpress, Shanghai, China) in 0.1 M KOH at 60 °C, as previously described [10,13,14]. Isorhamnetin was provided by ApexBio (Houston, TX, USA, purity ≥ 99.64%), and its chemical structural formula is shown in Figure S1. A stock solution of isorhamnetin was dissolved in dimethyl sulfoxide at a concentration of 10 mM and stored at −20 °C. Prior to use, different concentrations of isorhamnetin were prepared by dissolving it in the medium to achieve the appropriate dosage.

### 2.2. Cell Culture

The bovine endometrial epithelial cell line (bEECs) was purchased from Yaji Biological (Shanghai, China). Following thawing, bEECs were cultured in complete DMEM/F12 (Hyclone, Logan, CA, USA) culture medium. For identification purposes, the cells were immunoblotted with antibodies against cytokeratin 18 (Figure S2), which serves as an epithelial cell marker. After 2 to 3 passages, the cells were seeded into culture plates containing DMEM/F12 with 10% FBS, and the medium was replaced every 24 h. To evaluate the effects of varying NEFA concentrations on bEECs, the cells were cultured in DMEM/F12 medium supplemented with 0.11% fatty acid-free bovine serum albumin (BSA, Solebao, Beijing, China) and 0.1% insulin–transferrin–selenium (ITS, Sigma-Aldrich, Saint Louis, MO, USA). NEFA was applied for 24 h to cells at 0, 0.3, 0.6, or 1.2 mM. To assess the pharmacological effects of isorhamnetin on NEFA-treated cells, the cells were pretreated with isorhamnetin at concentrations of 1, 2.5, 5, or 10 µM for 12 h, followed by exposure to 1.2 mM NEFA for 24 h in serum-free DMEM/F12 medium (containing 0.11% BSA and 0.1% ITS).

### 2.3. Immunofluorescence

Cytokeratin 18 immunofluorescence staining was used to assess the purity of bEECs. Fixed cells underwent treatment with 4% paraformaldehyde for a duration of 30 min. Afterwards, they were subjected to permeabilization using 0.1% Triton X-100, which was maintained for a period of 1 h. Following washing with PBS, anti-cytokeratin 18 (Abcam, Cambridge, UK) was incubated overnight at 4 °C on the fixed cells. Following primary antibody incubation, the cells were treated with FITC-conjugated goat anti-rabbit IgG (Sigma-Aldrich, Saint Louis, MO, USA) for 45 min, followed by nuclear staining with 4′,6-diamidino-2-phenylindole (DAPI, Service bio, Wuhan, China) for 10 min. Fluorescence images were captured using a laser confocal microscope (Leica, Mannheim, Germany).

### 2.4. CCK-8 and EdU Assay

A CCK-8 kit (Dojindo Laboratories, Kumamoto, Japan) was used to measure cell viability according to the manufacturer’s instructions. For the EdU incorporation assay, cell proliferation was assessed using a 5-ethynyl-2′-deoxyuridine (EdU) assay kit (Med Chem Express, Shanghai, China) following the manufacturer’s instructions. Details are provided in Supplementary Materials and Methods.

### 2.5. Cell Cycle and Apoptosis Assay

After incubation with the indicated treatment, cell cycle analysis was performed using the Cell Cycle Kit (KeyGEN BioTECH, Nanjing, China). After fixation overnight at 4 °C in 70% ethanol, the cells were stained following the kit instructions, and the results were detected via flow cytometry. Apoptosis was assessed using the Annexin V-FITC Apoptosis Detection Kit (KeyGEN BioTECH, Nanjing, China). Briefly, drug-treated cells were harvested and resuspended in 500 μL of binding buffer containing the appropriate reagents. Subsequently, 5 μL of propidium iodide (PI) and 5 μL of Annexin V-FITC were sequentially added and mixed thoroughly. The cell suspension was incubated for 15 min at room temperature in the dark. Samples were then analyzed by flow cytometry, with data processed using FlowJo software (FlowJo, Version 10.4).

### 2.6. Triglyceride Measurement

Following treatment, the cells were digested with trypsin and subsequently collected by centrifugation. The concentrations of intracellular triglycerides were determined using a Triglyceride Assay Kit (Applygen Technologies Inc., Beijing, China) following the manufacturer’s instructions. The results were normalized to the concentration of protein. A BCA Protein Assay Kit (HY CEZMBIO, Wuhan, China) was used to measure protein content.

### 2.7. Detection of ROS

ROS levels in bEECs were measured using an ROS detection kit (Beyotime, Shanghai, China). Cells were incubated with 2 mL of 10 μM DCFH-DA (diluted in serum-free medium) at 37 °C for 20 min in the dark. After incubation, the cells were washed three times with serum-free medium to remove any excess probes, collected, and analyzed for ROS production via flow cytometry.

### 2.8. Oil Red O and BODIPY Staining

Lipid droplet formation was assessed using Oil Red O (Solarbio, Beijing, China) and BODIPY493/503 (MedChemExpress, Shanghai, China) staining techniques. Details are provided in Supplementary Materials and Methods.

### 2.9. Enzyme-Linked Immunosorbent Assay (ELISA)

Cytokine production (IL-1β, IL-6, and TNF-α) in the collected supernatant was measured using ELISA kits (Enzyme-Linked Biosystems, Shanghai, China). The optical density was then determined at 450 nm with a multimode microplate reader (Tecan, Männedorf, Switzerland).

### 2.10. Western Blotting Assay

RIPA lysis buffer was employed for protein extraction from bEECs. Protein concentration was assessed using the BCA kit. Following the addition of loading buffer (Servicebio, Wuhan, China) and boiling for 10 min, equivalent amounts of protein (10–20 μg) were separated by electrophoresis on a 10–15% SDS-PAGE gel and transferred to a PVDF membrane (Millipore, Billerica, MA, USA). The PVDF membrane was blocked with 5% skim milk for 2 h and then incubated overnight at 4 °C with primary antibodies against PPAR-γ (1:1000, ZEN-BIOSCIENCE, Chengdu, China), cleaved caspase-3 (1:1000, Abmart, Shanghai, China), BAX (1:1000, Affinity Biosciences, Liyang City, China), CyclinD (1:1000, Affinity Biosciences, Liyang City, China), p38 (1:1000, Affinity Biosciences, Liyang City, China), phospho-p38 (Thr180/Tyr182, 1:1000, Affinity Biosciences, Liyang City, China), phospho-JNK (Thr183/Tyr185, 1:1000, Abmart, Shanghai, China), JNK (1:1000, Abmart, Shanghai, China), phospho-ERK (Thr202/Tyr204, 1:1000, Abmart, Shanghai, China), ERK (1:1000, Abclone, Wuhan, China) and GAPDH (1:1000, Servicebio, Wuhan, China). After washing with TBST, the membrane was incubated with goat anti-rabbit/mouse IgG (H + L)-HRP (1:10,000, Abclone, Wuhan, China) at room temperature for 2 h. Following three washes with TBST, the protein bands were detected by an ECL developer (Biosharp, Anhui, China) and chemiluminescence imager (Tanon, Shanghai, China).

### 2.11. RNA-Seq and Transcriptome Analysis

Transcriptome sequences were obtained from three samples each of the control and 1.2 mM NEFA treatment groups. Differential gene expression analysis was performed using DESeq2 (v1.38.3), identifying genes with |log2FoldChange| > 1 and a *p*-value < 0.05 as differentially expressed genes (DEGs). A significant enrichment in GO and KEGG pathways was deemed significant by *p*-values ≤ 0.05 based on hypergeometric distribution tests. Details are provided in Supplementary Materials and Methods.

### 2.12. RNA Isolation and Quantitative Real-Time PCR

Total RNA isolated from cells was performed following the instructions of the total RNA extraction kit (Vazyme, Nanjing, China). Details are provided in Supplementary Materials and Methods.

### 2.13. Statistical Analysis

Data were analyzed and plotted by GraphPad (Version 6.0) and presented as mean ± standard error of the mean (SEM). The experiments were performed in three replicates (*n* = 3). For multiple comparisons, one-way ANOVA was conducted followed by Tukey’s post-hoc test.

## 3. Results

### 3.1. NEFA Inhibits Cell Viability and Induces Apoptosis in bEECs

**Figure 1:**
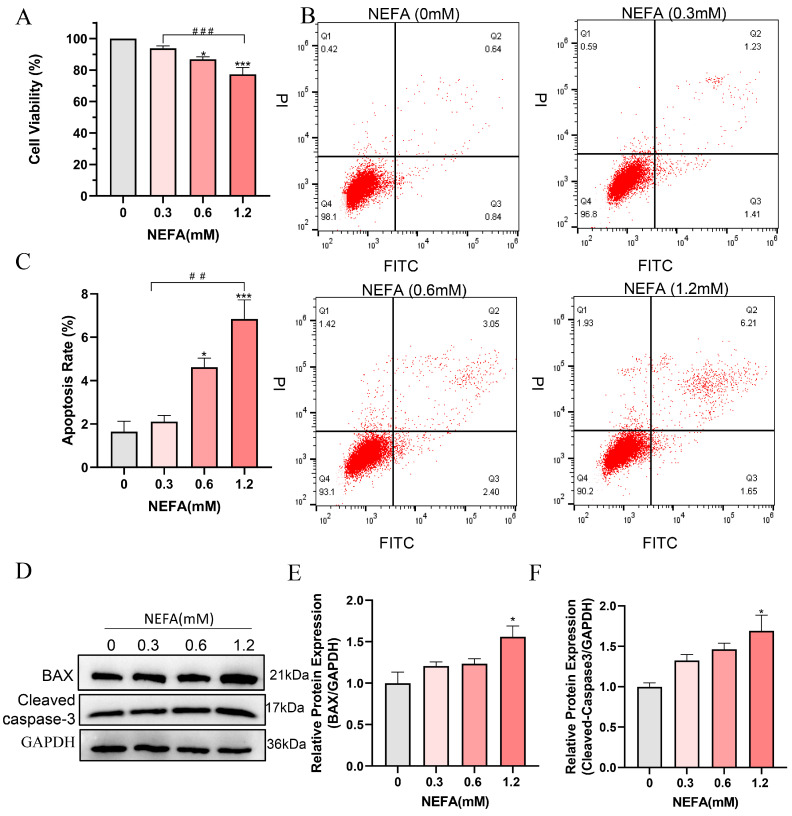
NEFA decreases cell proliferation and induces apoptosis in bEECs. BEECs were exposed to NEFA at the different concentrations (0, 0.3, 0.6, 1.2 mM) for 24 h. (**A**) Cell viability of bEECs was assessed using the CCK-8 assay. (**B**) Flow cytometry results illustrating the apoptotic rates of bEECs under various treatment conditions. (**C**) Quantification was presented by the ratio of apoptotic cells/total cells. (**D**) Western blot analysis of BAX and cleaved caspase3 in the different treatments. (**E**) Quantification of normalized protein expression intensity of BAX. (**F**) Quantification of normalized protein expression intensity of cleaved caspase-3. The experiments were performed in three replicates (*n* = 3). * *p* < 0.05, *** *p* < 0.001 indicate statistically significant differences between the treatment group and the control group;, ## *p* < 0.01, ### *p* < 0.001 indicate statistically significant differences between different treatment groups. No marking indicates no significant difference.

To determine whether NEFA induces cytotoxicity in bEECs, its effect on cell viability was examined. The results indicated that high concentrations of NEFA significantly reduced cell viability compared to the control group. Of note, a dosage of 1.2 mM of NEFA led to a decrease in cell viability to 77.3% (Figure 1A). Flow cytometry analysis revealed a dose-dependent increase in apoptosis rates following NEFA treatment (Figure 1B,C). High concentration NEFA treated cells showed higher levels of cleaved caspase-3 and the pro-apoptotic protein BAX compared to the control group (Figure 1D–F). These findings indicate that excess NEFA inhibits the cell viability and induces the apoptosis of bEECs.

### 3.2. NEFA Causes Lipid Accumulation in bEECs

**Figure 2:**
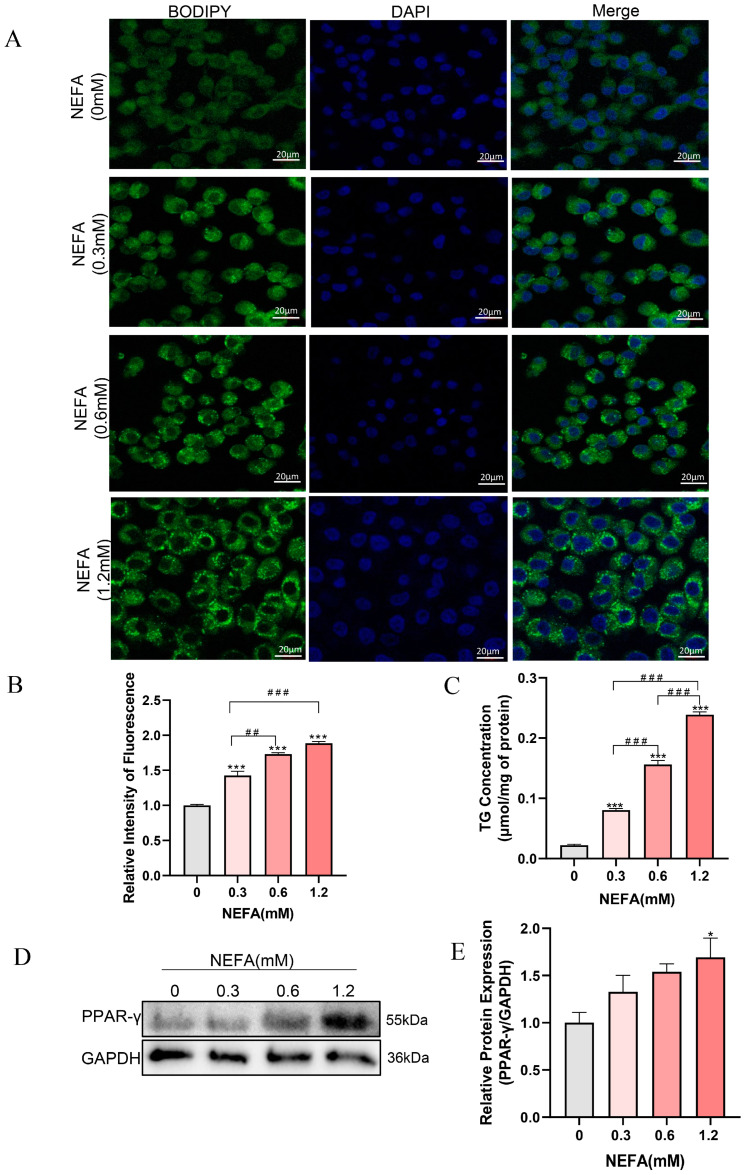
NEFA induces lipid accumulation in bEECs. BEECs were exposed to NEFA at different concentrations (0, 0.3, 0.6, 1.2 mM) for 24 h. (**A**) Representative BODIPY staining images of bEECs; scale bar: 20 μm. (**B**) Comparison of relative BODIPY fluorescence intensity in bEECs. (**C**) Intercellular TG content in bEECs. (**D**) Western blot analysis of PPAR-γ expression across various treatment groups. (**E**) Quantification of normalized protein expression intensity of PPAR-γ. The experiments were performed in three replicates (*n* = 3). * *p* < 0.05, *** *p* < 0.001 indicate statistically significant differences between the treatment group and the control group; ## *p* < 0.01, ### *p* < 0.001 indicate statistically significant differences between different treatment groups. No marking indicates no significant difference.

NEFA has been demonstrated to induce excessive lipid accumulation in various non-adipose cells of dairy cows, resulting in cellular dysfunction and even cell death [13,30]. To investigate whether NEFA can cause lipid accumulation in bEECs, the cells were stained with BODIPY (green) after treatment with various concentrations of NEFA for 24 h. As shown in Figure 2A,B, lipid accumulation within bEECs increased gradually with the increasing NEFA concentrations. Consistent with the BODIPY staining data, intercellular TG content in the various concentrations of NEFA-treated groups was markedly enhanced compared with the control group (Figure 2C). The maximum intercellular TG content was observed in 1.2 mM NEFA treatment group, which was 10-fold higher than that in the control group. In addition, Western blot results showed that NEFA significantly upregulated PPAR-γ protein expression, a key regulator of adipogenesis (Figure 2D,E). These findings indicate that NEFA induces lipid accumulation in bEECs.

### 3.3. NEFA Induces ROS Accumulation and the Release of Inflammatory Factors in bEECs

**Figure 3:**
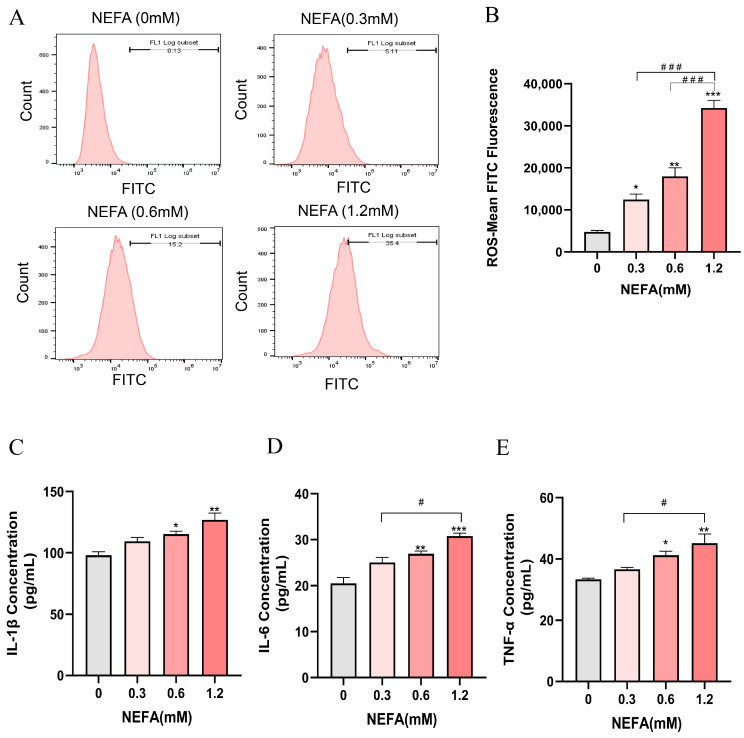
NEFA stimulates ROS accumulation and the release of inflammatory factors in bEECs. BEECs were exposed to NEFA at the different concentrations (0, 0.3, 0.6, 1.2 mM) for 24 h. (**A**) The level of ROS of bEECs were detected by flow cytometry. (**B**) Quantification of the level of ROS of bEECs. (**C**) IL-1β levels in the supernatant of bEECs treated with different concentrations of NEFA. (**D**) IL-6 levels in the supernatant of bEECs treated with different concentrations of NEFA. (**E**) TNF-α levels in the supernatant of bEECs treated with different concentrations of NEFA. The experiments were performed in three replicates (*n* = 3). * *p* < 0.05, ** *p* < 0.01, *** *p* < 0.001 indicate statistically significant differences between the treatment group and the control group; # *p* < 0.05, ### *p* < 0.001 indicate statistically significant differences between different treatment groups. No marking indicates no significant difference.

The intracellular ROS levels in bEECs exhibited a gradually increase with increasing NEFA concentrations, showing significant elevation in the treatment groups of 0.3, 0.6, and 1.2 mM NEFA when compared to the control group (Figure 3A,B). Subsequently, an ELISA assay was performed to investigate the concentrations of inflammatory cytokines, including IL-1β, IL-6, and TNF-α. The results indicated that as NEFA concentration increased, there was a marked rise in the release of pro-inflammatory cytokines IL-1β, IL-6, and TNF-α, which was significantly enhanced only in the groups treated with 0.6 mM and 1.2 mM NEFA when compared to the control group (Figure 3C–E). These findings suggest that excessive NEFA induces oxidative stress and inflammation in bEECs.

### 3.4. NEFA Alters a Variety of Biological Processes and Signaling Pathways in bEECs

**Figure 4:**
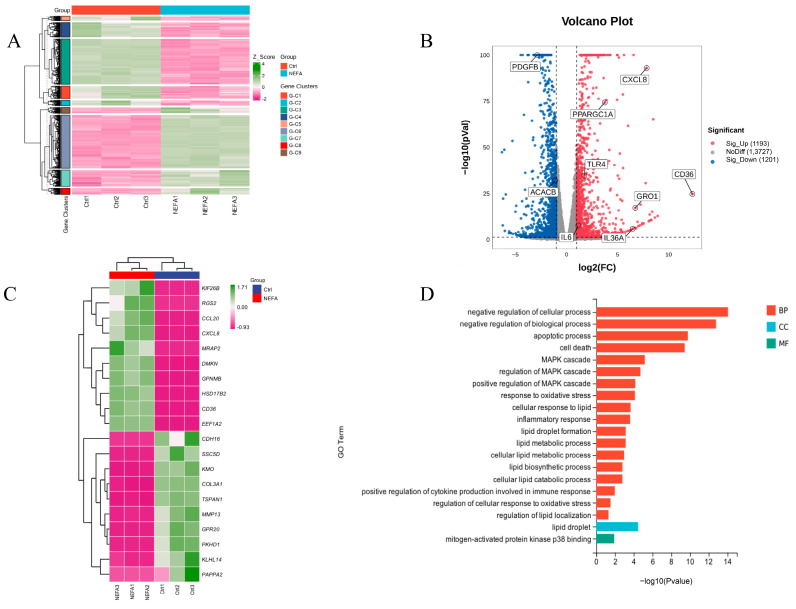

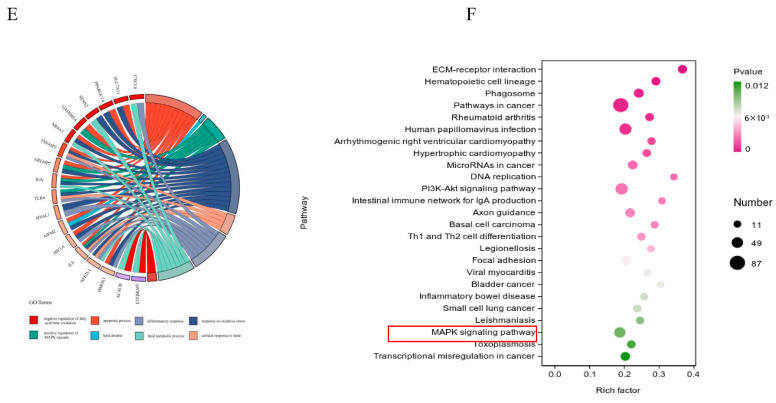
The results of RNA-seq data. BEECs were treated with 1.2 mM NEFA for 24 h. (**A**) The heatmap shows the expression of DEGs between the control group and NEFA group. (**B**) Volcano-plot representation of DEGs in control group and NEFA group. (**C**) The top 20 DEGs with the maximal fold changes (up- and downregulated) induced by NEFA. (**D**) Bar chart of the GO analysis of significant DEGs. (**E**) Chord diagram of the biological process analysis of significant DEGs. (**F**) Top 25 KEGG pathways with the highest enrichment; the red box represents the pathways we validated in subsequent experiments.

To further investigate the mechanisms behind NEFA-induced apoptosis, lipid accumulation, oxidative stress, and inflammation in bEECs, we next performed RNA sequencing (RNA-seq) analysis on cells after treatment with 1.2 mM NEFA. Heat map representation of these genes shows differential expression between the NEFA group and control group (Figure 4A). Compared to the control group, a total of 2394 DEGs were identified after NEFA treatment, including 1193 upregulated and 1201 downregulated genes (Figure 4B). The top 20 DEGs with the maximal fold changes (up- and downregulated) induced by NEFA are presented in Figure 4C. Several genes from DEGs were selected for RT-qPCR validation analysis, such as *CD36*, *CXCL8*, *IL-36*, *GRO1*, and *PDGFB* (Figure S3). A total of 1758 significant GO-BP terms, 154 significant GO-CC terms, and 251 significant GO-MF terms were identified. The significantly enriched GO terms mainly included the apoptotic process, cell death, MAPK cascade, response to oxidative stress, lipid droplet formation, and inflammatory response (Figure 4D). Chord diagrams revealed functional interconnections between multiple key biological processes and critical genes (Figure 4E). The scatter plot of KEGG pathway enrichment analysis revealed that the top 25 enrichment pathways primarily comprised the PI3K-Akt signaling pathway, MAPK signaling pathway, and ECM–receptor interaction (Figure 4F).

### 3.5. NEFA Activates MAPK Signaling Pathway in bEECs

**Figure 5:**
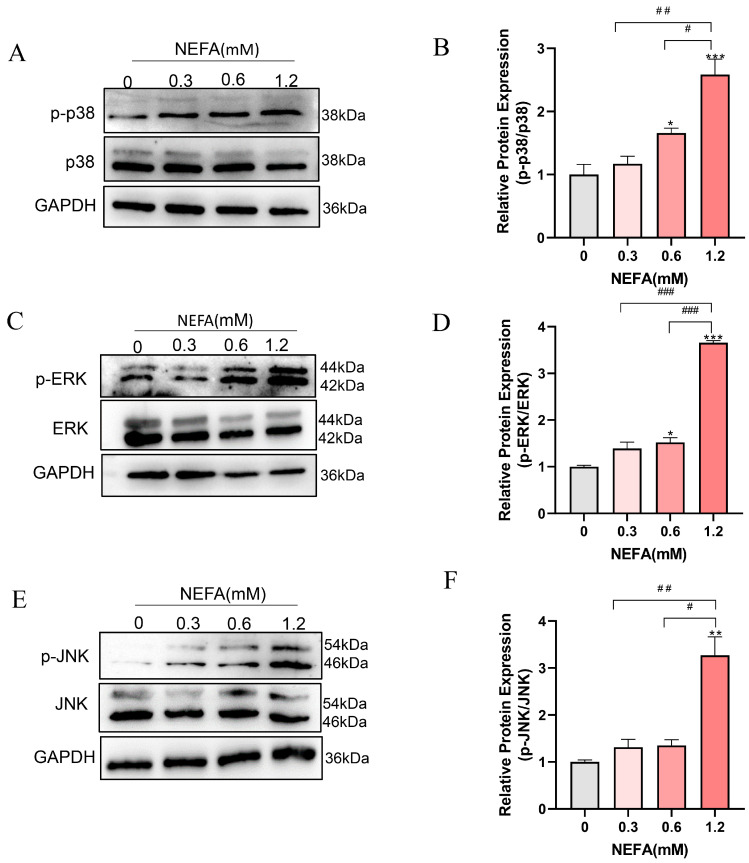
NEFA activates MAPK signaling pathway in bEECs. BEECs were exposed to NEFA at different concentrations (0, 0.3, 0.6, 1.2 mM) for 24 h. (**A**) Ex-pression levels of phosphorylated p38 and p38 in bEECs treated with varying concentrations of NEFA. (**B**) Quantification of the ratio of p-p38/p38. (**C**) Expression levels of phosphorylated ERK and ERK in bEECs treated with varying concentrations of NEFA. (**D**) Quantification of the ratio of p-ERK/ERK. (**E**) Expression levels of phosphorylated JNK and JNK in bEECs treated with varying concentrations of NEFA. (**F**) Quantification of the ratio of p-JNK/ JNK. The experiments were performed in three replicates (*n* = 3). * p < 0.05, ** p < 0.01, *** p < 0.001 indicate statistically significant differences between the treatment group and the control group; *# p* < 0.05, *## p* < 0.01, *### p* < 0.001 indicate statistically significant differences between different treatment groups. No marking indicates no significant difference.

Given that both GO and KEGG pathway analyses enriched the MAPK signaling pathway, which is closely linked to inflammation, cellular growth, and oxidative stress [31], we speculated that the MAPK signaling pathway may be involved in the adverse effects of NEFA on bEECs. Therefore, we further determined the expression changes in MAPK pathways including ERK, p38, and JNK by Western blot. As shown in Figure 5A–D, the phosphorylation levels of ERK and p38 were markedly activated in 0.6 mM and 1.2 mM NEFA-stimulated bEECs when compared to control, whereas the JNK phosphorylation was significantly activated in 1.2 mM NEFA-treated bEECs (Figure 5E,F). These findings demonstrate that excess NEFA can activate the MAPK signaling pathway in bEECs.

### 3.6. Isorhamnetin Promotes the Proliferation of bEECs

**Figure 6:**
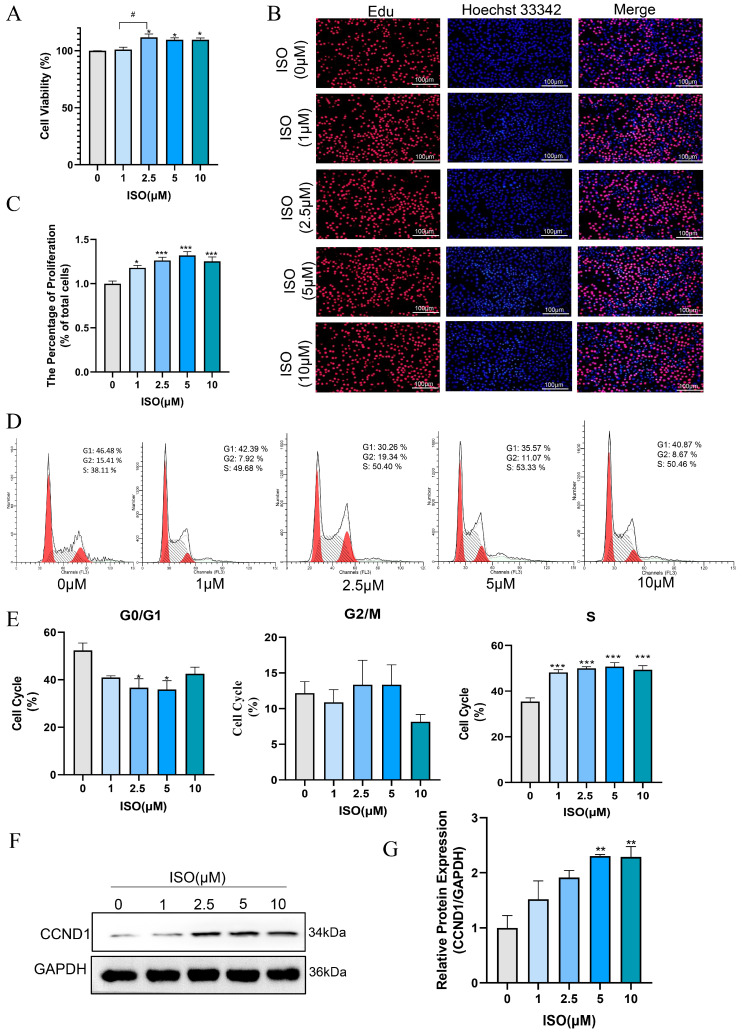
Isorhamnetin increases bEEC proliferation. BEECs were treated with varying concentrations of ISO (0, 1, 2.5, 5, and 10 μM) for 24 h. (**A**) Cell viability of bEECs was assessed using the CCK-8 assay. (**B**) Image of bEECs stained with EdU. EdU stained red and nuclei stained blue. Scale bar: 100 μm. (**C**) The bar chart displays the percentage of EdU-positive cells. (**D**) Flow cytometry results illustrating cell cycle distribution in the control and ISO-treated groups (1, 2.5, 5, and 10 μM). (**E**) The bar chart illustrates the cell cycle phase distributions (G0/G1, G2/M, and S) of bEECs across the various treatment groups. (**F**) Representative images of Western blot analysis of CCND1 in the different treatments. (**G**) Quantification of CCND1 protein levels in the different treatments. The experiments were performed in three replicates (*n* = 3). * *p* < 0.05, ** *p* < 0.01, *** *p* < 0.001 indicate statistically significant differences between the treatment group and the control group; # *p* < 0.05, indicate statistically significant differences between different treatment groups. No marking indicates no significant difference.

Isorhamnetin has been confirmed to possess multiple effects, such as anti-inflammatory, antioxidant, and anti-apoptotic properties [19,32]. In this study, we evaluated the effect of ISO on the proliferation and cell cycle of bEECs after treatment with various concentrations of ISO for 24 h. The results of CCK8 assay indicated that 2.5 μM, 5 μM and 10 μM isorhamnetin markedly raised the cell viability of bEECs when compared to the control group (Figure 6A). The quantification of EdU staining results showed a marked increase in the percentage of EdU-incorporating cells following treatment with 1, 2.5, 5, and 10 μM isorhamnetin (Figure 6B,C). In addition, flow cytometry analysis revealed that 1, 2.5, 5, and 10 μM isorhamnetin significantly enhanced the ratio of bEECs in the S-phase of the cell cycle (Figure 6D,E). Concurrently, western blot results revealed that the protein expression of Cyclin D significantly increased at 5μM and 10 μM isorhamnetin compared to the control group (Figure 6F,G). These findings indicate that isorhamnetin has a positive impact on the proliferation of bEECs.

### 3.7. Isorhamnetin Attenuates NEFA-Induced Apoptosis in bEECs

**Figure 7:**
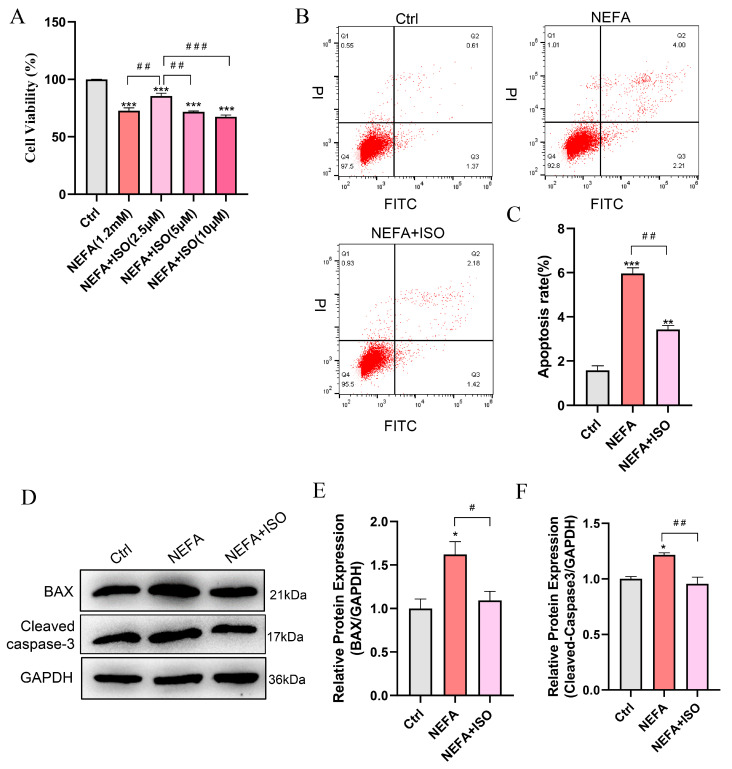
Isorhamnetin attenuates NEFA-induced apoptosis in bEECs. BEECs were pretreated with or without ISO at different concentrations (2.5, 5, 10 μM) or only 2.5 μM for 12 h and subsequently treated with NEFA (1.2 mM) for 24 h. (**A**) Cell viability of bEECs was assessed using the CCK-8 assay. (**B**) Flow cytometry results illustrating the apoptotic rates of bEECs under various treatment conditions. (**C**) Quantification was presented by the ratio of apoptotic cells/total cells. (**D**) Western blot analysis of BAX and cleaved caspase-3 in the different treatments. (**E**) Quantification of BAX protein levels in the different treatments. (**F**) Quantification of cleaved caspase3 protein levels in the different treatments. The experiments were performed in three replicates (*n* = 3). * *p* < 0.05, ** *p* < 0.01, *** *p* < 0.001 indicate statistically significant differences between the treatment group and the control group; # *p* < 0.05, ## *p* < 0.01, ### *p* < 0.001 indicate statistically significant differences between different treatment groups. No marking indicates no significant difference.

To explore the protective effect of ISO on NEFA-induced apoptosis in bEECs, the cells were pretreated with varying concentrations of ISO for 12 h, then exposed to 1.2 mM NEFA for 24 h. The CCK-8 assay indicated that the cell viability of bEECs in the combined group of 2.5 μM ISO and NEFA increased by 12.9% compared to the NEFA alone treatment group (Figure 7A). Consequently, a concentration of 2.5 µM ISO was selected for subsequent experiments. Furthermore, isorhamnetin (2.5 μM) remarkably reduced the cell apoptosis rate compared to the NEFA alone treatment group (Figure 7B,C), which was accompanied by a significant decrease in the apoptotic biomarkers BAX and cleaved caspase-3 (Figure 7D–F). Taken together, these results indicate that ISO ameliorates the impaired cell viability and apoptosis induced by NEFA in bEECs.

### 3.8. Isorhamnetin Mitigates NEFA-Induced Lipid Accumulation in bEECs

**Figure 8:**
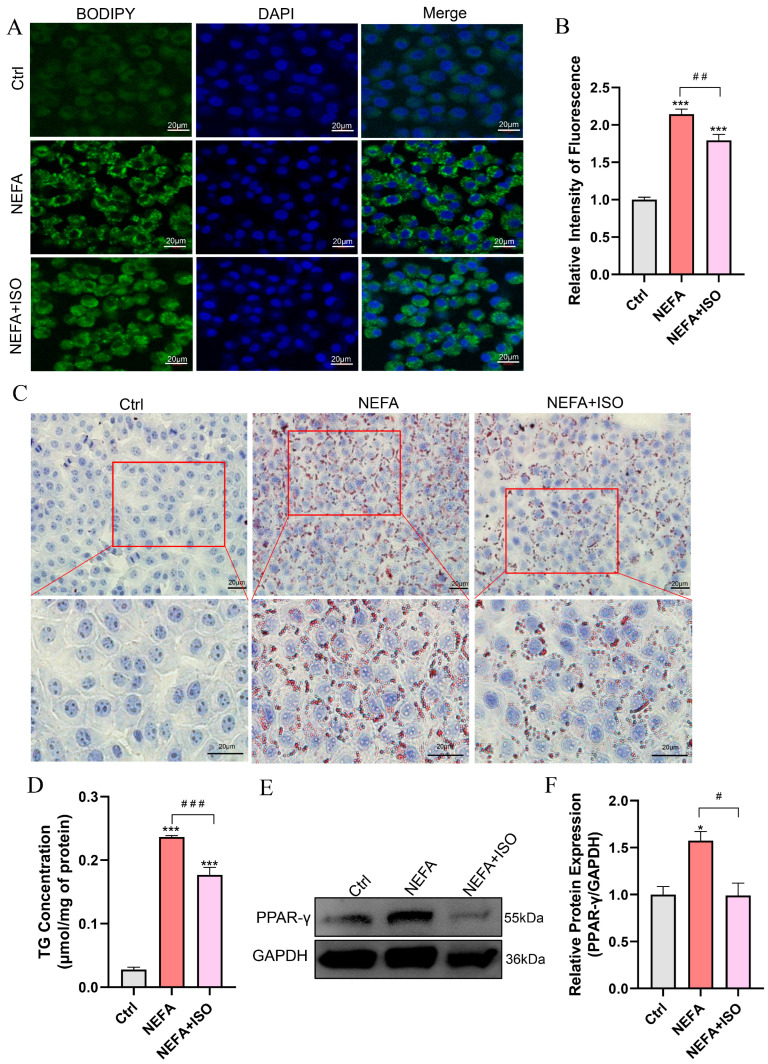
Isorhamnetin reduces lipid accumulation in NEFA-induced bEECs. BEECs were pretreated with or without ISO at a concentration of 2.5 µM for 12 h, followed by exposure to NEFA (1.2 mM) for an additional 24 h. (**A**) Representative BODIPY staining images of bEECs; scale bar: 20 μm. (**B**) Comparison of relative BODIPY fluorescence intensity in bEECs. (**C**) Representative Oil Red O staining images of bEECs; scale bar: 20 μm. (**D**) Intercellular TG content in bEECs. (**E**) Western blot analysis of PPAR-γ expression across various treatment groups. (**F**) Quantification of normalized protein expression intensity of PPAR-γ. The experiments were performed in three replicates (*n* = 3). * *p* < 0.05, *** *p* < 0.001 indicate statistically significant differences between the treatment group and the control group; # *p* < 0.05, ## *p* < 0.01, ### *p* < 0.001 indicate statistically significant differences between different treatment groups. No marking indicates no significant difference.

BODIPY and Oil Red O staining revealed that lipid droplet (LD) content was remarkably reduced in the isorhamnetin pretreatment plus NEFA group compared to the NEFA alone treatment group (Figure 8A–C). Similarly, NEFA alone treatment increased the TG content to 8.47 times that of control group, while pretreatment with isorhamnetin (2.5 µM) recovered it to 6.33 times that of control group (Figure 8D). In addition, ISO significantly reduced the protein expression of PPAR-γ compared to the NEFA alone treatment group (Figure 8E,F). These findings suggest that ISO alleviates lipid accumulation caused by NEFA in bEECs.

### 3.9. Isorhamnetin Decreases NEFA-Induced ROS Accumulation and the Release of Inflammatory Factors in bEECs

**Figure 9:**
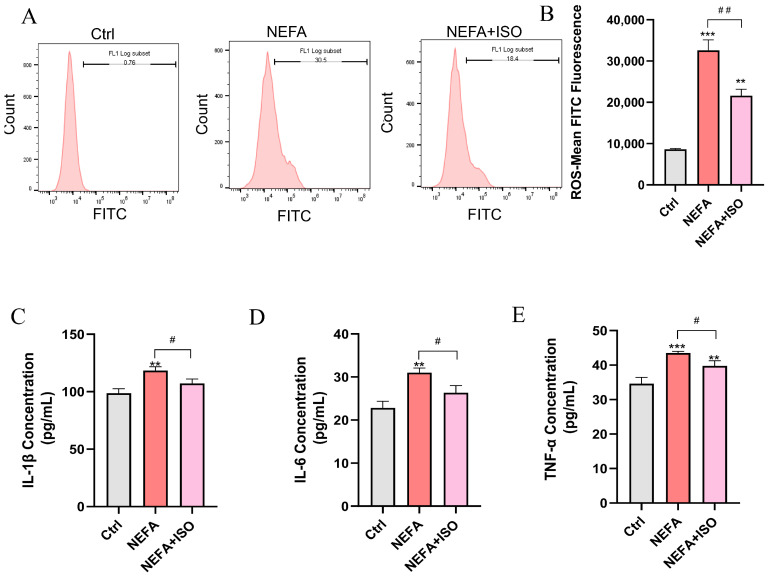
Isorhamnetin decreases NEFA-induced ROS accumulation and the release of inflammatory factors in bEECs. BEECs were pretreated with or without ISO at a concentration of 2.5 µM for 12 h, followed by exposure to NEFA (1.2 mM) for an additional 24 h. (**A**) The level of ROS in bEECs was detected by flow cytometry. (**B**) Quantification of the level of ROS in bEECs. (**C**) IL-1β levels in the supernatant of bEECs. (**D**) IL-6 levels in the supernatant of bEECs. (**E**) TNF-α levels in the supernatant of bEECs. The experiments were performed in three replicates (*n* = 3). ** *p* < 0.01, *** *p* < 0.001 indicate statistically significant differences between the treatment group and the control group; # *p* < 0.05, ## *p* < 0.01, indicate statistically significant differences between different treatment groups. No marking indicates no significant difference.

ISO is reported to exhibit potent anti-inflammatory and antioxidant properties [19], but whether it can reduce ROS and the release of inflammatory factors in bEECs remains unknown. As illustrated in Figure 9A,B, ISO effectively attenuated the levels of ROS induced by NEFA treatment. Furthermore, ELISA results showed that ISO could remarkedly decreased the levels of IL-1β, IL-6, and TNF-α in cell culture supernatant and reverse the inflammatory response induced by NEFA (Figure 9C–E). Our findings reveal that ISO effectively reduces ROS generation and suppresses the inflammatory response in NEFA-treated bEECs.

### 3.10. Isorhamnetin Reduces NEFA-Induced Activation of MAPK Signaling Pathway

**Figure 10:**
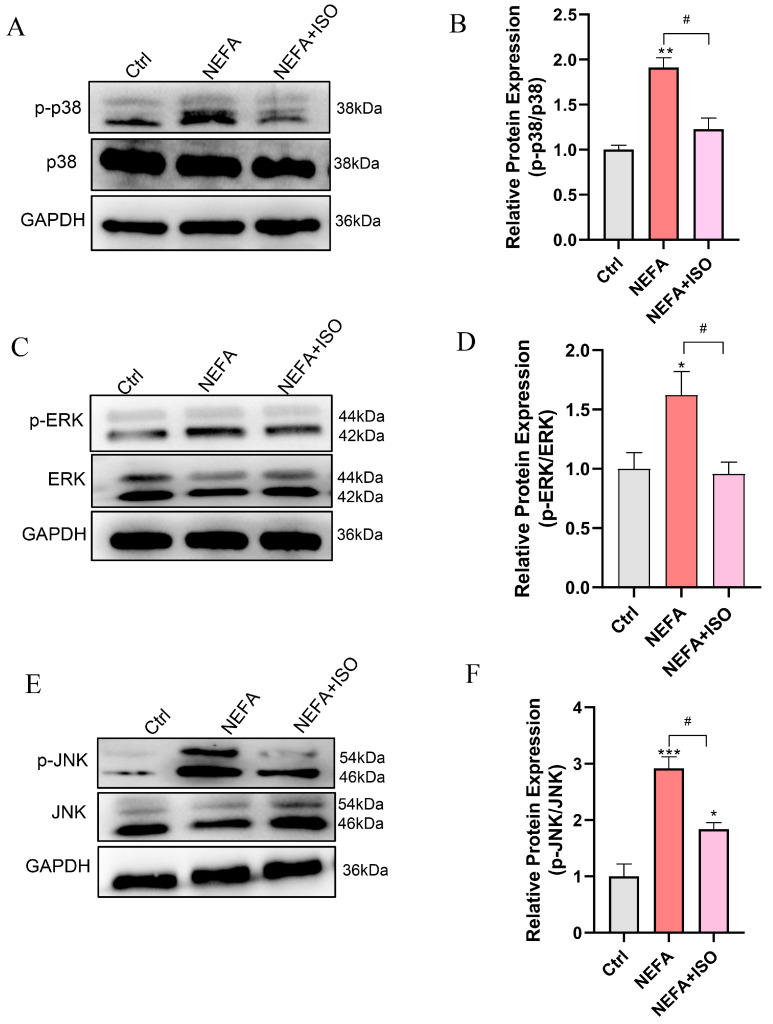
Isorhamnetin reduces NEFA-induced activation of MAPK pathway. BEECs were pretreated with or without ISO at a concentration of 2.5 µM for 12 h, followed by exposure to NEFA (1.2 mM) for an additional 24 h. (**A**) Western blot analysis of p38 and p-p38 in the different treatments. (**B**) Quantification of the ratio of p-p38/p38. (**C**) Western blot analysis of ERK and p-PERK in the different treatments. (**D**) Quantification of the ratio of p-ERK/ERK. (**E**) Western blot analysis of JNK and p-JNK in the different treatments. (**F**) Quantification of the ratio of p-JNK/JNK. The experiments were performed in three replicates (*n* = 3). * *p* < 0.05, ** *p* < 0.01, *** *p* < 0.001 indicate statistically significant differences between the treatment group and the control group; # *p* < 0.05, indicates statistically significant differences between different treatment groups. No marking indicates no significant difference.

To further investigate the therapeutic impact of ISO on NEFA-induced cellular injury, the MAPK signaling pathway was examined through Western blot. The results demonstrated that pretreatment with 2.5 μM ISO remarkedly reduced the phosphorylation levels of ERK, JNK, and p38 relative to the NEFA alone treatment group (Figure 10A–F). These findings suggest that ISO may attenuate the NEFA-induced bEECs damage through the MAPK signaling pathway.

## 4. Discussion

High blood concentrations of NEFA are considered as a significant risk factor for ketosis in dairy cows during the transition period. In this study, we observed that elevated levels of NEFA lead to damage in bEECs, as evidenced by decreased cell viability, activation of apoptosis, lipid accumulation, ROS generation, and the release of inflammatory factors. Alleviating these NEFA-induced negative effects is crucial for maintaining endometrial health, establishing pregnancy, and improving embryo development in cattle. Isorhamnetin, a flavonoid present in various medicinal plants, has been demonstrated in numerous studies to possess considerable anti-inflammatory and anti-obesity properties. Our findings confirm that ISO may mitigate NEFA-induced damage in bEECs by modulating the MAPK signaling pathways. These findings provide novel evidence that ISO could serve as a therapeutic agent to protect the endometrium of dairy cows during a transitional period.

Cows with higher serum NEFA concentrations tended to suffer from subclinical endometritis [33]; however, the precise regulatory effects of NEFA on endometrium cells remain to be elucidated. In this study, we evaluated the effects of different concentrations of NEFA (0.3, 0.6, and 1.2 mM) on the proliferation, apoptosis, lipid accumulation, ROS generation, and inflammation in bEECs. These varying concentrations of NEFA may correspond to different clinical conditions. Additionally, we primarily focused on shorter exposure times (12 h and 24 h), as our objective was to investigate the acute effects of NEFA exposure, which are relevant to the pathophysiological conditions of clinical ketosis. However, we also recognize that exploring longer exposure durations, especially at lower NEFA concentrations, could provide valuable insights into the chronic cytotoxic effects and represents an important direction for future research. This study found that NEFA reduced bEEC viability in a dose-dependent manner, accompanied by a dose-dependent increase in cell apoptosis. These findings are consistent with previous studies conducted by Chankeaw’s team [34]. The apoptotic processes induced by NEFA have been reported through various mechanisms, including the induction of endoplasmic reticulum (ER) stress [35] and the activation of caspase-3 [36]. Here, we demonstrated that NEFA exerts pro-apoptotic effects by upregulating the expression of BAX and cleaved caspase-3, suggesting that NEFA causes mitochondrial dysfunction in bEECs. At the cellular level, previous studies have reported that the negative effects on cell viability and apoptosis may be related to the lipotoxicity induced by NEFA [13,34]. Our research further demonstrated that NEFA treatment increased the BODIPY fluorescence intensity and intracellular TG content, indicating NEFA induces lipid accumulation in bEECs. Concurrently, this effect is strongly supported by our RNA-seq data, which demonstrate that DEGs are mainly enriched in lipid-related biological processes, including cellular response to lipid, lipid droplet formation, lipid biosynthetic process, and lipid metabolic process. Notably, NEFA-stimulated lipid accumulation may arise from the expression upregulation of PPAR-γ, a key regulator responsible for triglyceride formation and storage in lipid droplets [37]. Recent studies have shown that an overload of lipids can induce oxidative stress [38]. Several studies have demonstrated that elevated NEFA levels are linked to increased mitochondrial ROS production and oxidative stress across various cell types, including bovine hepatocytes and granulosa cells [18,39]. Similarly, our experiments demonstrated that ROS levels increased in a NEFA dose-dependent manner in bEECs. In addition, ROS production is often associated with the occurrence of cellular inflammation [40]. Consistently, we observed that excessive NEFA stimulated the secretion of inflammatory factors IL-1β, TNF-α, and IL-6 in bEECs. This observation was further confirmed by GO enrichment analysis.

Recently, isorhamnetin, as a flavonoid active compound, has been widely studied. ISO has been reported to protect porcine oocytes from apoptosis, oxidative stress, and endoplasmic reticulum stress induced by zearalenone through the PI3K/AKT signaling pathway [41]. ISO pretreatment can reduce the loss of mitochondrial membrane potential, improve cellular morphological damage, and inhibit H_2_O_2_-induced apoptosis in HaCaT cells [42]. According to the published data, isorhamnetin raised the ratio of S-phase cells in response to GC proliferation by increasing Cyclin family protein expression [32]. Consistent with these findings, we observed that isorhamnetin treatment increased the ratio of cells in S-phase and Cyclin D expression significantly. Meanwhile, we also proved that ISO treatment promoted the proliferation of bEECs, as indicated by EdU-positive cells. These findings suggest that ISO is beneficial for bEEC growth. Besides the pro-proliferation effect, ISO is a novel antagonist of PPAR-γ that inhibits adipocyte differentiation induced by the PPAR-γ agonist rosiglitazone, thereby obstructing obesity development and alleviating hepatic steatosis caused by high-fat diet and leptin deficiency [27]. Lee et al. demonstrated that low concentrations of ISO (0.1, 0.5, 1, and 10 μM) can inhibit adipogenesis and promote mitochondrial biogenesis in 3T3-L1 cells [43]. These results suggest that isorhamnetin may have a protective effect against NEFA-induced damage and lipid accumulation in bEECs.

Interestingly, we found that 2.5 µM ISO is more effective than 5 μM or 10 μM in protecting bEECs from the effects of NEFA. Based on these beneficial effects, we further investigated the potential impact of 2.5 µM ISO in alleviating damage to bEECs induced by NEFA. Compared to the NEFA alone treatment group, the addition of ISO significantly enhanced cell viability and reduced the apoptosis rate by downregulating the expression of BAX and cleaved caspase-3 in bEECs. Furthermore, our findings demonstrated that ISO significantly alleviated oxidative stress and the inflammatory response induced by NEFA in bEECs, as evidence by a decrease in the production of ROS, IL-1β, IL-6, and TNF-α. Our results are consistent with former studies showing that isorhamnetin can alleviate oxidative stress and inflammation in paracetamol-induced liver damage [44] and heat stroke-affected lung injury [45]. As the antagonist of PPAR-γ, ISO expectedly downregulated the expression of PPAR-γ, thus reducing lipid accumulation in NEFA-treated bEECs. Notably, the protective effect of ISO observed in bEECs in this study may be a subsequent effect of reduced lipid accumulation. For instance, isorhamnetin-mediated reduction in lipid accumulation may attributed to decreased ROS production and inflammatory response, thereby alleviating cell apoptosis induced by NEFA. To our knowledge, this study is the first to report the inhibitory effect of isorhamnetin on lipotoxicity in bEECs.

The MAPK pathways relay, amplify, and integrate signals from various stimuli, triggering physiological responses such as cellular proliferation, differentiation, development, inflammatory reactions, and apoptosis in mammalian cells [46]. It has been proved that NEFA induced hepatocyte apoptosis mediating via the ROS-ERK/JNK signaling pathway and oxidative stress [39]. Likewise, NEFA increased reactive oxygen species (ROS) levels in bovine mammary epithelial cells by activating the MAPK signaling pathway [47]. In the current study, KEGG and GO analyses revealed that the MAPK signaling pathways were significantly enriched in NEFA treatment cells, indicating MAPK may play a significant role in NEFA-induced damage to bEECs. Subsequently, Western blot results confirmed a dose-dependent increase in the phosphorylation levels of JNK, ERK, and p38 following NEFA treatment. Of note, isorhamnetin attenuates the NEFA-induced activation of the MAPK signaling pathway in bEECs, as evidenced by the reduction in the phosphorylation expression of JNK, ERK, and p38. These findings align with previous observations that ISO inhibits LPS-induced phosphorylation of ERK and JNK in vivo by modulating the MAPK signaling pathways [48].

## 5. Conclusions

**Figure 11:**
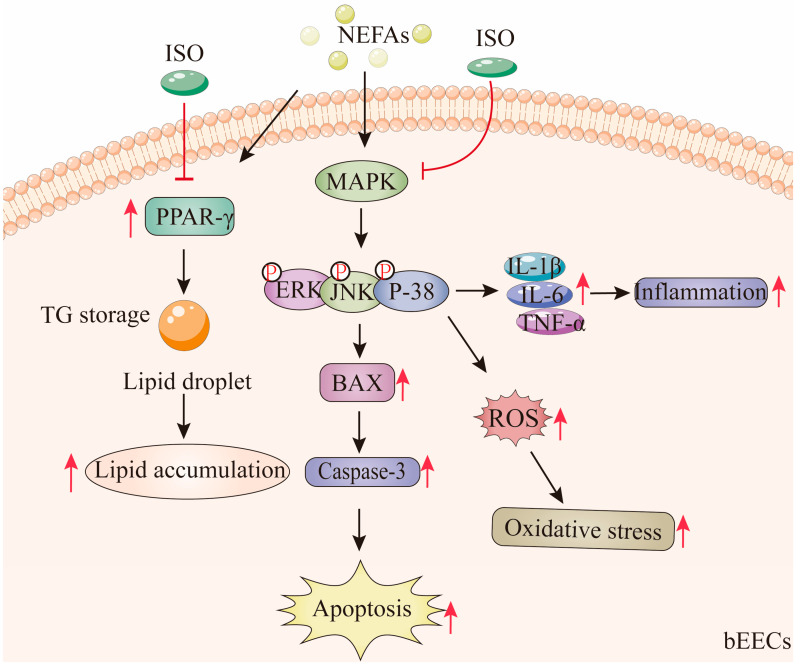
Isorhamnetin ameliorates NEFA-induced damage in bovine endometrial epithelial cells via inhibiting MAPK signaling pathway. High concentrations of NEFA induce lipid accumulation in bEECs and trigger apoptosis, oxidative stress, and inflammatory responses via activation of the MAPK pathway. Notably, ISO, as a PPAR-γ antagonist, alleviate NEFA-induced lipid accumulation in bEECs by downregulating PPAR-γ. In addition, ISO mitigates apoptosis, oxidative stress, and inflammation in bEECs by attenuating NEFA-activated MAPK signaling. The red “T” arrow repre-sents “inhibition”; the black arrow represents “promotion”.

According to these findings, medium and high concentrations of NEFA induce lipid accumulation in bEECs and trigger apoptosis, oxidative stress, and inflammatory responses via activation of the MAPK signaling pathways, including p38, ERK, and JNK. Notably, ISO, as a PPAR-γ antagonist, may alleviate NEFA-induced lipid accumulation in bEECs by downregulating the expression of PPAR-γ. In addition, we confirmed that ISO mitigates apoptosis, oxidative stress, and inflammation in bEECs by attenuating NEFA-activated MAPK signaling (Figure 11). Collectively, these findings illuminate that isorhamnetin may be a promising candidate for pharmaceutical development or as part of a dietbased strategy to support bovine uterine health during the transition period.

## Data Availability

The RNA-seq data reported in this article are available in the NCBI database (Bioproject PRJNA1163307).

## Supplementary Materials

The following supporting information can be downloaded at: https://www.mdpi.com/article/10.3390/antiox14020156/s1, Figure S1: Chemical structure of isorhamnetin. Figure S2: Cytokeratin 18 (CK 18) immunofluorescence staining. Nuclei (blue) and CK18 (green); scale bar represents 10 μM. Figure S3: mRNA expression of CD36, CXCL8, IL-36, GRO1, and PDGFB.
