# Supplementary material for: Isorhamnetin Ameliorates Non-Esterified Fatty Acid-Induced Apoptosis, Lipid Accumulation, and Oxidative Stress in Bovine Endometrial Epithelial Cells via Inhibiting the MAPK Signaling Pathway
Source: Antioxidants (Basel). 2025 Jan 28;14(2):156. doi: 10.3390/antiox14020156 (PMC11852151; doi:10.3390/antiox14020156)
Supplement: Supplementary file 1 [file antioxidants-14-00156-s001.zip › antioxidants-3316517-supplementary.pdf]

Isorhamnetin Ameliorates Non-Esterified Fatty Acid-Induced Apoptosis, Lipid Accumulation, and Oxidative Stress in Bovine Endometrial Epithelial Cells via Inhibiting the MAPK Signaling Pathway

Haimiao Lv [1], Lijuan Liu [1], Wenna Zou [1], Ying Yang [1], Yuan Li [1], Shengji Yang [1], Aixin Liang [1,2,*] and Liguo Yang [1,2,*]

[1] Key Laboratory of Agricultural Animal Genetics, Breeding and Reproduction of Ministry of Education, College of Animal Science and Technology, Huazhong Agricultural University, Wuhan 430070, China

[2] National Center for International Research on Animal Genetics, Breeding and Reproduction, Huazhong Agricultural University, Wuhan 430070, China

* **Correspondence**:

E-mail: lax.pipi@mail.hzau.edu.cn (A.L.); ylg@mail.hzau.edu.cn (L.Y.)

Supplementary materials and methods

2.4. CCK-8 and EdU assay

Cell viability was measured using a CCK8 kit (Dojindo Laboratories, Kumamoto, Japan) according to the manufacturer's instructions. A total of 5 × 10^3 cells per well were seeded in 96-well plates and incubated at 37°C in a 5% CO_2 atmosphere. After incubating with indicated treatment for 24 h, 10 μL of CCK-8 solution was added to each well, and the plates were further incubated for 4 h at 37°C in 5% CO_2. The optical density was subsequently measured at 450 nm using a Tecan Spark multimode microplate reader (Tecan, Switzerland).

For the EdU incorporation assay, cell proliferation was assessed using a 5-ethynyl-2'-deoxyuridine (EdU) assay kit (Med Chem Express, Shanghai, China) following the manufacturer's instructions. Briefly, 24 h after transfection, the cells were exposed to 10 μM EdU for 2 h. Afterward, the cells were fixed with 4% paraformaldehyde and permeabilized with 0.5% Triton X-100. Subsequently, the cells were stained with Hoechst 33342 for 30 min. An inverted fluorescence microscope (Olympus, Tokyo, Japan) was used to detect all cells. The ratio of EdU-positive cells to the total number of cells was then calculated for analysis.

2.7. Oil Red O and BODIPY Staining

Lipid droplet formation was assessed using Oil Red O and BODIPY staining techniques. The Oil Red O staining was performed with an Oil Red O Kit (Solarbio, Beijing, China). Following treatment, the cells were washed three times with ice-cold PBS, fixed in 4% paraformaldehyde for 15 min at room temperature, and subsequently washed again with PBS. The cells were then incubated in 60% isopropanol for 10 s, then with Oil Red O for 30 min, and counterstained with hematoxylin before microscopy.

For BODIPY staining, cells were incubated with 2 μM BODIPY 493/503 (MedChemExpress, Shanghai, China) for 15 min at 37°C. Following incubation, the cells were placed on a horizontal shaker and washed with PBS every 5 min, repeating this process three times. Subsequently, the cells were stained with DAPI for 10 min, washed three more times, and images were captured using an

inverted confocal microscope (Leica, Mannheim, Germany).

2.10. RNA-Seq and Transcriptome Analysis

Transcriptome sequences were obtained from three samples each of the control and 1.2 mM NEFA treatment groups. Total RNA from NEFA-treated and untreated cells was extracted using an RNA extraction kit (Vazyme, Nanjing, China). RNA quality was assessed with a NanoDrop spectrophotometer (Thermo Fisher Scientific, Waltham, USA) and verified by RNase-free agarose gel electrophoresis. The RNA library was prepared with a NEBNext Ultra RNA library prep kit for Illumina (New England Biolabs Inc., Ipswich, MA, USA). The sequencing library was then sequenced on the NovaSeq 6000 platform (Illumina) at Shanghai Personal Biotechnology Co. Ltd. Reads were filtered using HISAT2 (v2.1.0) and mapped to the ARS-UCD1.2 Bos taurus genome (GCA_002263795.2). Differential gene expression analysis was performed using DESeq2 (v1.38.3), identifying genes with |log2FoldChange| > 1 and a P-value < 0.05 as differentially expressed genes (DEGs). GO and KEGG pathway enrichment analyses were conducted using hypergeometric distribution tests, with P-values ≤ 0.05 indicating significant enrichment.

2.11. RNA Isolation and Quantitative Real-Time PCR

Total RNA was isolated using a total RNA extraction kit (Vazyme, Nanjing, China). RNA purity was assessed by measuring the UV absorbance ratio at 260/280 nm with a K5500 Micro-Spectrophotometer. Subsequently, 1 μg of total RNA from each sample was reverse-transcribed into cDNA (Beijing Tiangen Biology Co., Ltd., Beijing, China) following the manufacturer's protocol. Quantitative reverse transcription PCR (qRT-PCR) was conducted using a 7500 Real-Time PCR System (Applied Biosystems) with an SYBR Green Plus reagent kit (Roche, Norwalk, USA). The PCR conditions were as follows: initial denaturation at 94 °C for 2min, followed by 35 cycles of 94 °C for 10s, 60 °C for 15s, and 72 °C for 30s, with a final extension at 72 °C for 5 min. Each cDNA sample was analyzed in triplicate from three independent experiments. β-actin was used as the reference gene for normalization, and mRNA expression levels were calculated using the $2^{-\Delta\Delta CT}$ method. Primer sequences are detailed in Table S1.

Table S1. List of real-time PCR primers used in this study.

Gene	Gene accessions no	Forward primer 5′ - 3′	Reverse primer 5′ - 3′
CD36	XM_059885389.1	CTCATTGCTGGTGCTGTCATT	CCTTGGCTAGATAACGAACTCTG
CXCL8	NM_173925.2	CCTCTTGTTCAATATGACTTCCA	GGCCCACTCTCAATAACTCTC
IL36A	XM_059891762.1	GGAGCCGGAGCTTTGTCT	CCCTGCCATTCTGGTCAT
GRO1	NM_175700.2	AACCGAAGTCATAGCCAGTCTCA	GGCACTAGCCTTGTTTAGCATC
PDGFB	XM_024992036.2	TGAGATCGTGCGGAAGAAGAAG	GAATGGTGCACCCGAGTTTGG
β-actin	NM_173979.3	CACGTGGAGGGGCCGGACTCATC	TAAAGACCTCTATGCCAACACAGT

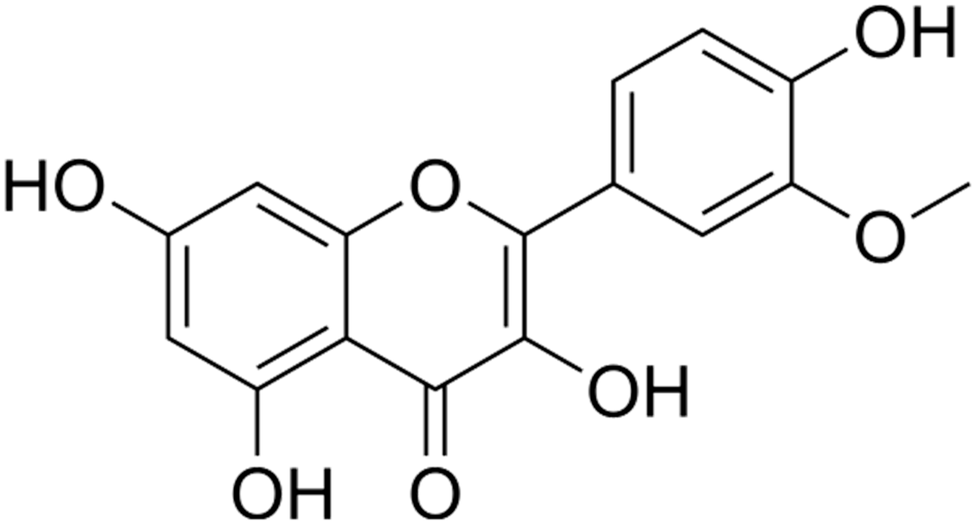

Figure S1. Chemical structure of isorhamnetin.

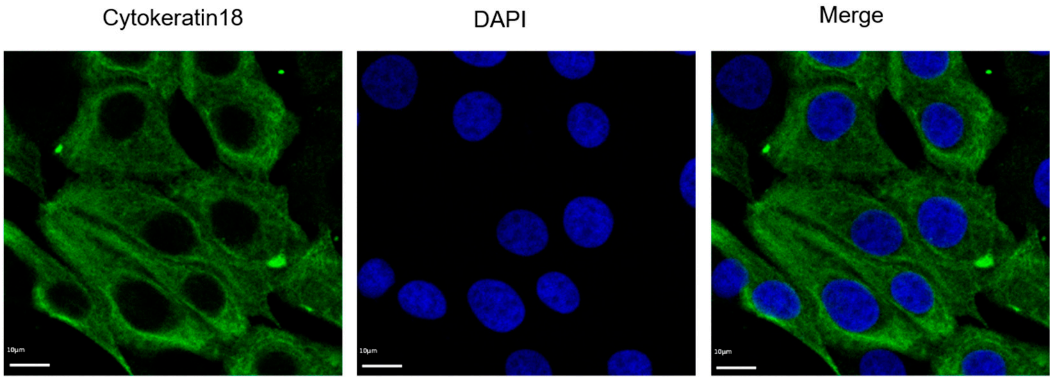

Figure S2. Cytokeratin 18 (CK 18) immunofluorescence staining. Nuclei (blue) and CK18 (green); scale bar represents 10 μM.

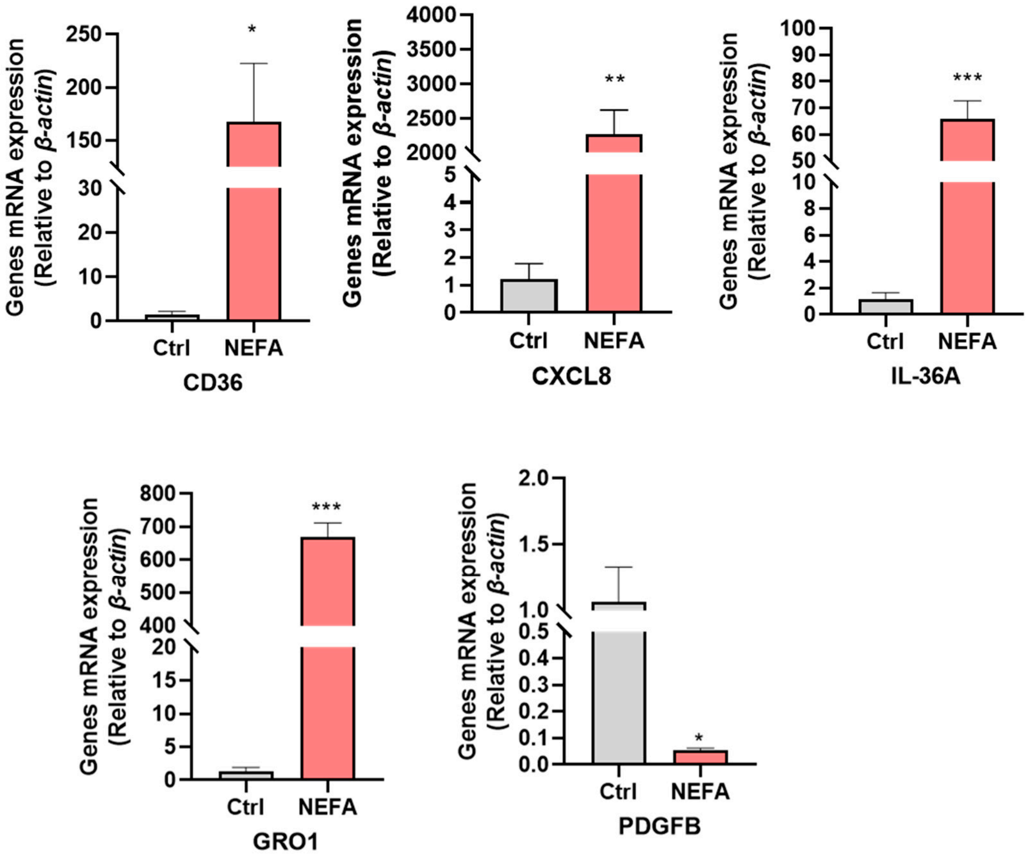

Figure S3. mRNA expression of CD36, CXCL8, IL-36, GRO1, and PDGFB.
